# Out of sight, hidden into the deep: Marine litter in the Mar del Plata Submarine Canyon (southwestern Atlantic) revealed by ROV survey

**DOI:** 10.1371/journal.pone.0357098

**Published:** 2026-09-16

**Authors:** Valeria Teso, Diego Urteaga, Graziella Bozzano, Gregorio Bigatti, Martín I. Brogger, Francisco Brusa, Rodrigo Calderón, Nadia Cerino, Ignacio L. Chiesa, María Agustina Conti, Cristina Damborenea, María Carla de Aranzamendi, Brenda L. Doti, Nahuel Farías, Jonathan N. Flores, Santiago Herrera, Ezequiel Mabragaña, Mariano Martinez, Florencia Matusevich, Adriana Menoret, Emiliano Ocampo, Leonel Pacheco, Guido Pastorino, Pablo Penchaszadeh, Emanuel Pereira, Renata M. Pertossi, Jessica Risaro, Noelia C. Sánchez, Javier H. Signorelli, Marcos Tatián, Johanna N. J. Weston, Daniel Lauretta

**Affiliations:** 1 Museo Argentino de Ciencias Naturales “Bernardino Rivadavia” (MACN), Buenos Aires, Argentina; 2 Grupo de Estudios del Mar Profundo de Argentina (GEMPA), Argentina; 3 Servicio de Hidrografía Naval (SHN), Buenos Aires, Argentina; 4 Instituto de Biología de Organismos Marinos (IBIOMAR, CCT-CONICET CENPAT), Puerto Madryn, Argentina; 5 Universidad Espíritu Santo, Ecuador; 6 Museo de La Plata, Facultad de Ciencias Naturales y Museo, UNLP (MLP), La Plata, Argentina; 7 Universidad de Buenos Aires, Facultad de Ciencias Exactas y Naturales, Departamento de Biodiversidad y Biología Experimental (DBBE), Buenos Aires, Argentina; 8 Prefectura Naval Argentina (PNA), Buenos Aires, Argentina; 9 Centro Austral de Investigaciones Científicas (CADIC), Ushuaia, Argentina; 10 Universidad Nacional de Córdoba (UNC), Facultad de Ciencias Exactas, Físicas y Naturales, Córdoba, Argentina; 11 Instituto de Diversidad y Ecología Animal (IDEA, CONICET-UNC), Córdoba, Argentina; 12 Universidad de Buenos Aires - CONICET, Instituto de Biodiversidad y Biología Experimental y Aplicada (IBBEA), Buenos Aires, Argentina; 13 Instituto de Investigaciones Marinas y Costeras (IIMyC, FCEyN-UNMDP/ CONICET), Mar del Plata, Argentina; 14 Lehigh University, Oceans Research Center, Bethlehem, United States of America; 15 Woods Hole Oceanographic Institution, Woods Hole, United States of America; University of Messina, ITALY

## Abstract

The deep sea is increasingly recognized as vulnerable to anthropogenic pressures, including marine litter, yet large geographic gaps remain in our understanding of litter distribution and ecological interactions. Submarine canyon systems can act as conduits and accumulation zones for anthropogenic material, but data from the southwestern Atlantic are scarce. Here, we present the first characterization of deep-sea marine macro-litter in the Mar del Plata Submarine Canyon (Argentina), based on 240 hours of remotely operated vehicle (ROV) video collected during 17 dives (878–3,820 m depth) aboard the R/V *Falkor (too)* in July-August 2025. Litter was observed during 10 dives, comprising 29 items distributed between 1,120 and 3,820 m. Plastic was the dominant material (27 items), followed by rubber (2 items). Most litter consisted of plastic sheets and bags, food wrappers, and commercial fishing-related gear, including lines, ropes, and cables. Litter was observed in multiple settings, including buried or degraded items, resting on the seabed, and trapped among rocks or drifting near the bottom. Most items were located within the canyon domain, forming localized clusters along the canyon axis and walls. Litter-fauna interactions were documented for seven items and included both sessile colonization and associations with mobile fauna, mainly on partially buried plastic items and entangled fishing-related gear. These patterns suggest that substrate stability and residence time may influence sessile colonization. The composition and distribution of debris indicate that maritime activities, particularly fisheries, are the most likely sources and further support the role of submarine canyons as accumulation zones for marine litter. This study provides critical baseline data in an understudied region and highlights the need for improved monitoring programs and management strategies for marine litter to reduce impacts in submarine canyon systems.

## Introduction

Long regarded as remote and largely insulated from human activities, the deep sea (depths >200 m) is now recognized as being increasingly affected by multiple anthropogenic pressures, including fishing, resource extraction, climate change, and marine pollution [1–3]. Among the most visible manifestations of this impact is the accumulation of anthropogenic litter, including plastics, which has been documented from the coastal and intertidal zones to some of the most remote deep-sea habitats worldwide [4–6]. Marine litter is defined as “any persistent, manufactured or processed solid material discarded, disposed of or abandoned in the marine and coastal environment” [7]. The seafloor and sediments act as major sinks for anthropogenic litter, often accumulating higher concentrations than in the overlying water column [8], with potential consequences for biodiversity and food-web functioning. Furthermore, the extremely slow degradation rates characteristic of the deep sea, combined with the long durability of industrial materials, promote the long-term preservation and accumulation of anthropogenic litter on the seafloor [1,4,5].

Submarine canyons have emerged as a hotspot for marine litter accumulation, with densities significantly exceeding those reported from adjacent continental slopes and continental shelves [9]. This pattern is largely driven by their complex geomorphology and hydrodynamic regimes, which facilitate the transport, concentration, and retention of materials originating from continental shelves and coastal areas [10–13]. Once in the canyon, benthic fauna can interact with the litter deposited on the seafloor through six major pathways: entanglement, ingestion, smothering, habitat provision, adaptive behaviour, and incidental encountering [14]. These interactions include direct physical impacts (e.g., entanglement, ingestion and smothering), behavioural responses to litter, encounters with debris during normal activities, and the use of litter as artificial habitat. By providing novel attachment surfaces, litter can promote colonisation and increase habitat heterogeneity, which potentially alters ecological interactions and benthic community composition [13,15]. In addition, abandoned, lost, or discarded fishing gear can continue to capture or entangle organisms through ghost fishing, resulting in prolonged impacts on marine fauna [16]. Collectively, these litter-fauna interactions highlight the ecological significance of marine macro-litter beyond its mere presence on the seafloor and emphasize the need for targeted investigations in submarine canyon systems, where high biodiversity and anthropogenic litter frequently converge.

Despite increasing global interest in deep-sea marine litter, research has been concentrated largely in the Northern Hemisphere, particularly in the Mediterranean Sea, the North Atlantic, and the Pacific Ocean. This Northern focus results in pronounced geographical biases in our current understanding of marine litter distribution and ecological impacts [1,4,6,17]. Marine litter has been documented in surface waters and the water column of the southwestern Atlantic, including reports of floating litter and its ingestion by marine megafauna [18,19]. Evidence of anthropogenic debris has also recently been reported from the Argentine deep sea, highlighting its accumulation across deep-water habitats [20]. However, the occurrence, distribution, and ecological implications of anthropogenic litter in deep-sea habitats of the region have received little attention in the scientific literature [3,21]. Within the southwestern Atlantic Ocean, the Mar del Plata Submarine Canyon (MPSC) is a biologically important feature that serves as a major conduit linking shelf and deep-sea ecosystems. To address knowledge gaps, we document the occurrence and distribution of deep-sea marine litter within the MPSC, based on video data collected during the first remotely operated vehicle (ROV) explorations of the canyon as part of the *Talud Continental IV* Expedition (2025).

### Study area

The MPSC (~38ºS) is one of the major geomorphological features along the Argentine continental margin. Located about 250 km off the coast, it is considered a land-detached canyon as no connection with the continental shelf exists [22]. The canyon head begins at a depth of about 900 m, while the characteristic V-shaped morphology is fully developed between 1,200 and 3,500 m depth [23,24]. The surveyed area extended from the upper continental slope (~780 m) to nearly 3,900 m depth.

The MPSC lies beneath the Brazil-Malvinas Confluence, a highly dynamic oceanographic region formed by the interaction between the warm, poleward-flowing Brazil Current and the cold, nutrient-rich Malvinas Current, which originates in the Drake Passage from the northernmost branch of the Antarctic Circumpolar Current and flows equatorward [25]. The Brazil-Malvinas Confluence is characterized by complex water-mass structures, high hydrodynamic variability, and enhanced biological productivity [26]. These processes make the MPSC an important pathway for sediment transport and organic matter from the continental shelf to the deep sea [23,27].

Deep-water fisheries have operated in the southwestern Atlantic for several decades, particularly in the southern latitudes of the Patagonian margin. The region supports commercially important resources including the Argentinean shortfinned squid *Illex argentinus*, Patagonian toothfish *Dissostichus eleginoides*, southern blue whiting *Micromesistius australis*, Patagonian grenadier *Macruronus* *magellanicus,* and pink cusk-eel *Genypterus blacodes*. These resources are targeted using bottom trawls and longline gear [28–33]. In contrast, deep-water fisheries in the northern Argentine Sea have been comparatively limited and are focused primarily on *G. blacodes*, which commonly aggregates within submarine canyons at the edge of the continental shelf and is caught there almost exclusively [24,34].

## Materials and Methods

**Fig 1:**
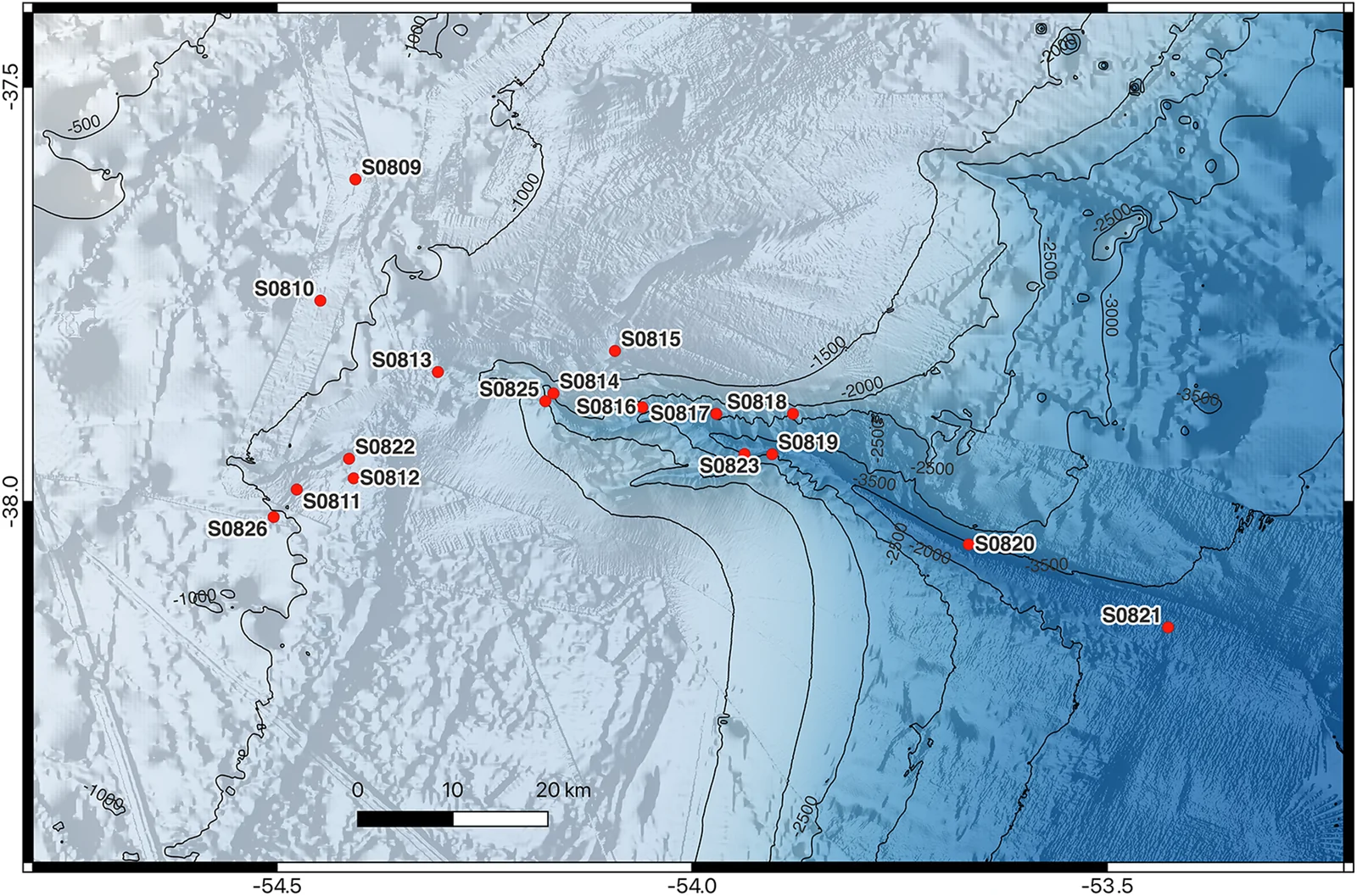
Bathymetry of the Mar del Plata Submarine Canyon study area (SW Atlantic) and ROV dive locations. Red dots represent the center of each dive track. The high-resolution bathymetry data were collected during the Fkt250712 expedition, whereas the low-resolution bathymetry data were obtained from the GEBCO_2026 Grid from the Global Multi-Resolution Topography synthesis. The figure was created using QGIS v. 3.26 [35].

A total of 17 dives were conducted aboard the R/V *Falkor (too)* during the Schmidt Ocean Institute (SOI) *Talud Continental IV* expedition (FKt250712) in July-August 2025 using the ROV *SuBastian* (S), which was equipped with high-definition (4K) cameras, two hydraulic manipulators, and bioboxes for specimen collection. The dives spanned depths of 878–3,820 m and generated approximately 240 hours of video footage (Fig 1). All videos are available online through the Schmidt Ocean Institute’s YouTube channel (https://www.youtube.com/@SchmidtOcean; dives S0809 to S0826), and on the Grupo de Estudios del Mar Profundo de Argentina YouTube channel (https://www.youtube.com/@GEMPA. ARGENTINA). The use and analysis of these data complied with the terms and conditions established by the Schmidt Ocean Institute.

High-resolution bathymetry of the study area was collected using the hull-mounted Kongsberg EM124 and EM712 deep-sea echosounders. Raw bathymetric data were processed and cleaned onboard with Qimera software (QPS, v2.5) to generate a 60 × 60 m digital elevation model (DEM), which was exported as a GeoTIFF and imported into QGIS v3.4 for spatial analysis and map production. In areas where high-resolution bathymetry data was unavailable, data from the Global Multi-Resolution Topography synthesis was used.

### Video analysis

All litter on the seafloor and water column observations made during ROV operations were logged in real-time using SeaLog V2.2.2 software [36,37]. In select cases, large litter items were recovered with the ROV manipulators to allow direct examination of associated fauna that were not observable or confidently identifiable from video imagery alone.

Following the expedition, video footage was systematically reviewed to identify anthropogenic litter present on the seafloor and in the water column. For each litter item recorded, latitude, longitude, depth, substrate type (soft-bottom, hard-bottom, or mixed), litter-fauna interaction (e.g., colonization, entanglement, or shelter), and corresponding video reference were compiled (Table 1, S1 Table). Only items that could be confidently identified as anthropogenic were included. Anthropogenic litter was distinguished from natural materials based on characteristics such as artificial coloration, printed patterns, manufactured textures, and regular geometric shapes. Size estimates were obtained using the ROV’s paired laser scaling system, which projects two green laser points spaced 10 cm apart onto the seafloor. When both laser points were visible, a scale bar was added to the image to facilitate measurements. Benthic organisms associated with litter items were identified to the lowest possible taxonomic level from imagery and, when available, from collected specimens.

**Table 1:** Classification of marine litter items recorded during ROV dives in the Mar del Plata Submarine Canyon, including dive identification code, sampling site (CM: Conturite moat; NCW: Northern canyon wall; SCW: Southern canyon wall; CV: canyon valley), water depth (m), litter category and type, type of L-F interaction, substrate, and associated phyla.

Item	Dive	Site	Depth	Litter Category (MSFD)	Litter Type	Interaction type	Substrate	Associated Phylum
1	S0812	CM	1120	Plastic	Bag	Habitat provision	Mixed	Cnidaria
2	S0814	NCW	2105	Plastic	Wrapper	–	Water column	
3	S0814	NCW	2109	Plastic	Food packaging	–	Soft bottom	
4	S0814	NCW	2108	Plastic	Other	Habitat provision	Soft bottom	Arthropoda, Cnidaria
5	S0814	NCW	2108	Plastic	Food packaging	–	Soft bottom	
6	S0814	NCW	2105	Plastic	Bag	–	Soft bottom	
7	S0814	NCW	2101	Plastic	Other	–	Soft bottom	
8	S0814	NCW	2054	Plastic	Rope	Habitat provision	Soft bottom	Annelida
9	S0814	NCW	2047	Plastic	Wrapper	Encountering	Soft bottom	
10	S0814	NCW	1616	Plastic	Filament	Habitat provision	Mixed	Cnidaria
11	S0815	NCW	1468	Plastic	Filament	–	Mixed	
12	S0816	NCW	1920	Plastic	Food packaging	–	Mixed	
13	S0816	NCW	1913	Plastic	Bag	Encountering	Mixed	Arthropoda
14	S0816	NCW	2598	Rubber	Clothes	–	Soft bottom	
15	S0816	NCW	1764	Plastic	Other	Habitat provision and encountering	Soft bottom	Chordata, Echinodermata
16	S0818	NCW	2165	Plastic	Other	–	Soft bottom	
17	S0819	SCW	3243	Plastic	Cup	–	Mixed with big boulders	
18	S0819	SCW	3243	Plastic	Bag	Encountering	Mixed with big boulders	Echinodermata
19	S0819	SCW	3231	Plastic	Food packaging	–	Mixed with big boulders	
20	S0819	SCW	3110	Plastic	Bag	–	Mixed	
21	S0819	SCW	3078	Rubber	Clothes	–	Hard bottom with sediments	
22	S0820	NCW	3739	Plastic	Filament	–	Soft bottom	
23	S0820	NCW	3739	Plastic	Bag	–	Soft bottom	
24	S0820	NCW	3722	Plastic	Bag	–	Soft bottom	
25	S0820	NCW	3720	Plastic	Bag	–	Soft bottom	
26	S0820	NCW	3709	Plastic	Other	–	Soft bottom	
27	S0821	CV	3814	Plastic	Wrapper	–	Soft bottom	
28	S0823	SCW	3075	Plastic	Other	–	Soft bottom	
29	S0825	SCW	1949	Plastic	Rope	–	Hard bottom	

## Results

**Fig 2:**
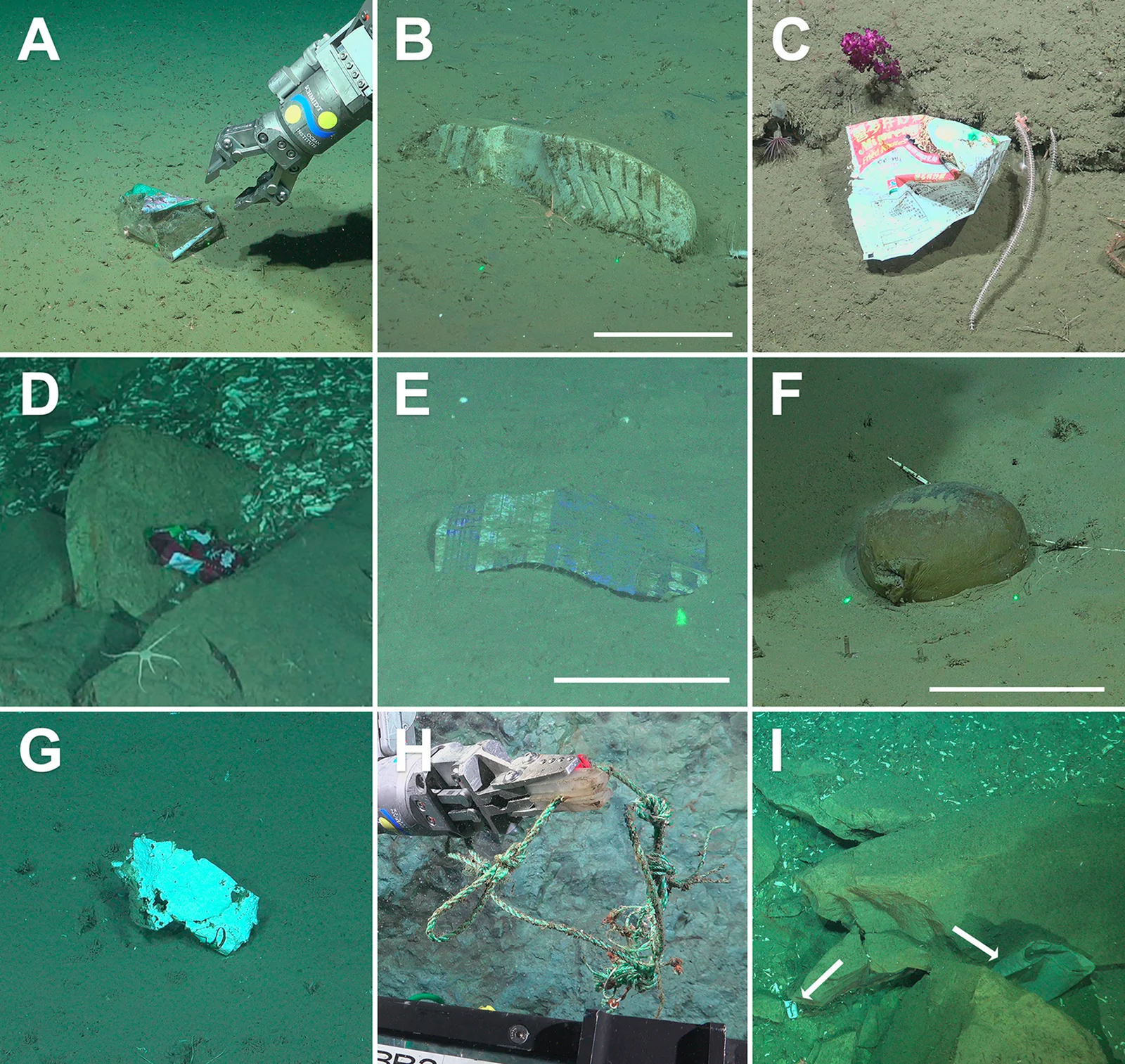
Examples of deep-sea litter found in the Mar del Plata Submarine Canyon. **Dive identification codes (e.g., S0814, S0819) correspond to ROV SuBastian dives.** (A) Medium-sized plastic wrapper (S0814 - event 3), 2,109 m. (B) Rubber boot (S0816 - event 14), 2,598 m. (C) Food wrapper (S0816 - event 12), 1,920 m. (D) Snack wrapper (S0819 - event 19), 3,231 m. (E) Tissue paper wrapper (S0821 - event 27), 3,814 m. (F) Sediment-filled plastic bag (S0820 - event 23), 3,739 m. (G) Highly degraded plastic fragment (S0820 - event 26), 3,709 m. (H) Nylon cord with a hook (S0825 - event 29), 1,949 m. (I) White plastic cup and bag (arrows) (S0819 - items 17-18), 3,243 m. Scale bars: 10 cm. All images courtesy of Schmidt Ocean Institute/ ROV SuBastian (CC BY 4.0), expedition FKt250712.

Deep-sea litter was observed during 10 of the 17 dives (58.8%). A total of 29 items were documented between 1,120 and 3,820 m. Plastic accounted for the vast majority of observations (27 items), followed by rubber (2 items) (Fig 2, Table 1, S1 Table). Plastic litter consisted primarily of food wrappers and unidentified fragments, followed by plastic bags and fishing-related gear. Several items were partially buried in the sediment, whereas others rested on the seafloor or were observed drifting close to the bottom (Table 1).

### Spatial distribution and composition of marine litter within the canyon

**Fig 3:**
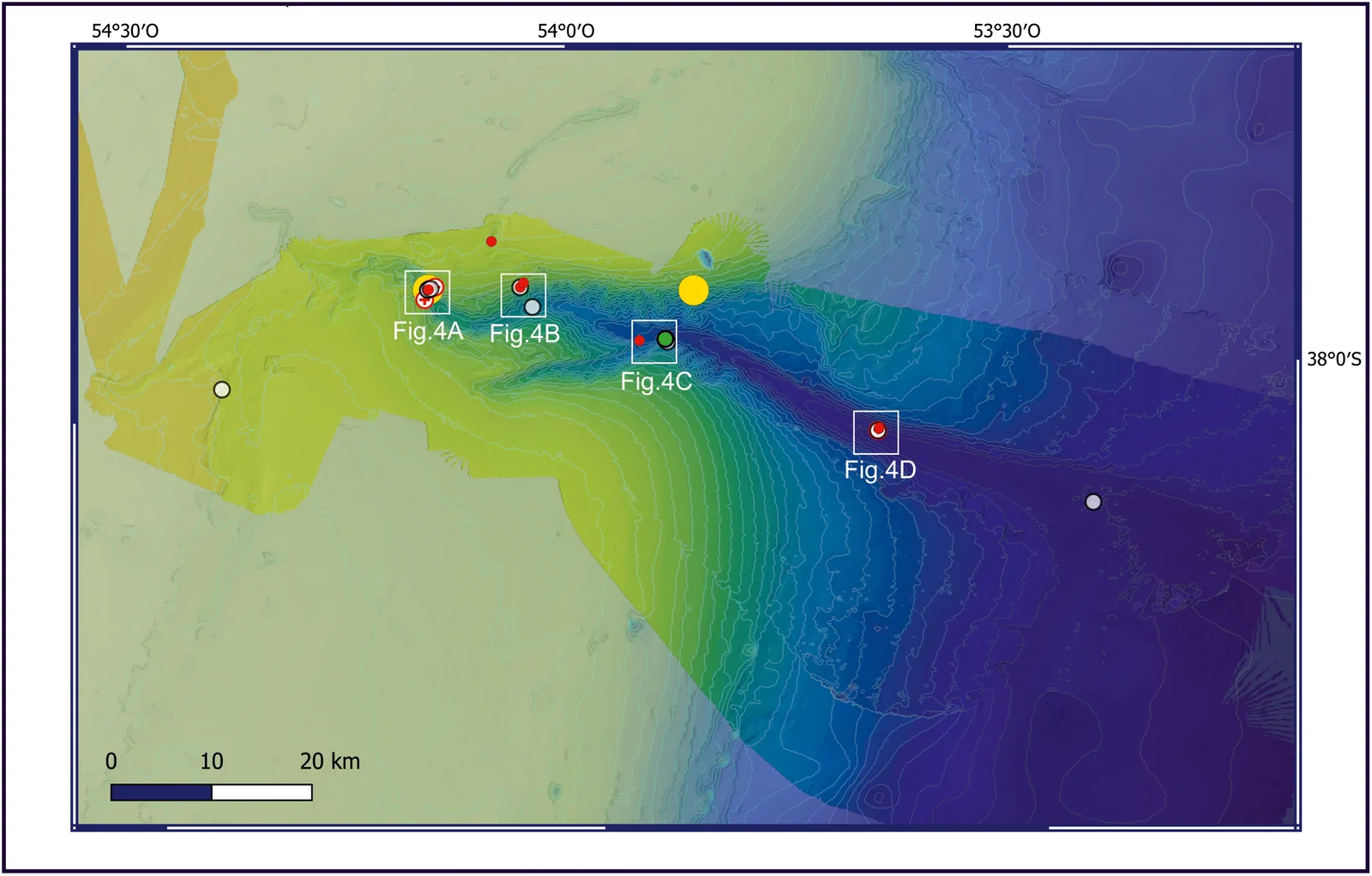
Overview of the distribution and types of marine litter in the Mar del Plata Submarine Canyon: buried or degraded litter (red dots), litter lying on the seabed (white transparent dots), fishing gear (red cross), litter trapped between rocks (green dots), and litter drifting in the water column (yellow dots).

**Fig 4:**
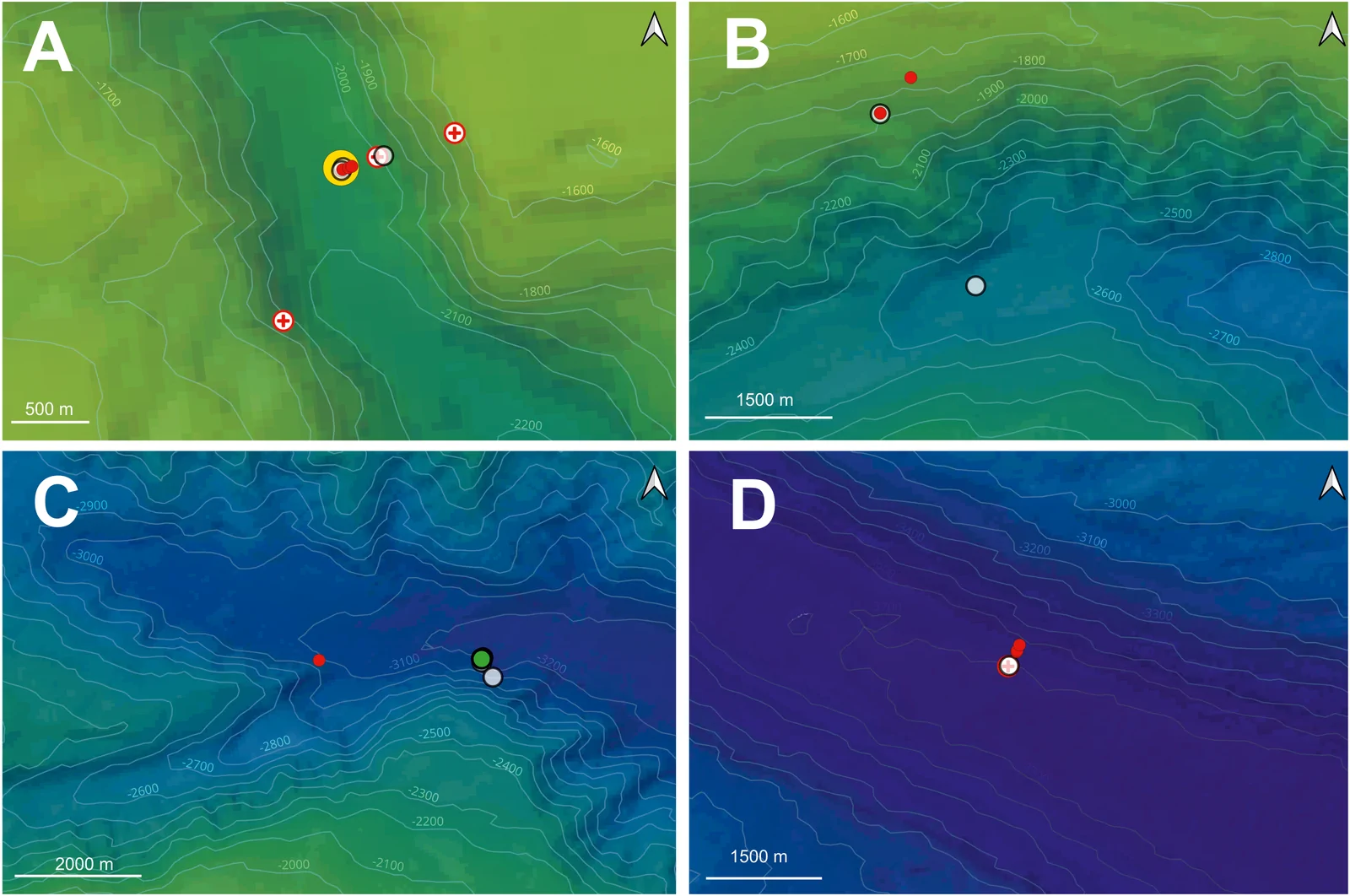
Close-up view of some of the areas affected by marine litter in the Mar del Plata Submarine Canyon. A: Upper sector, cluster of 10 items found along dives S0814 and S0818; B: Upper sector, cluster of 4 items; C: Lower sector, cluster of 5 items; D: Deepest sector, cluster of 5 items. Buried or degraded litter (red dots), litter lying on the seabed (white transparent dots), fishing gear (red cross), litter trapped between rocks (green dots), and litter drifting in the water column (yellow dots).

The 29 marine litter items observed during the expedition were classified into five categories: buried or degraded items (n = 10), items lying on the seabed (n = 10), fishing gear (n = 4), items trapped among rocks (n = 3), and items drifting in the water column (n = 2). Most litter was observed within the canyon domain. Exceptions included a plastic bag found in the contourite moat south of the canyon, a drifting plastic item sighted near the rim of the northern wall at a depth of 2,100 m, and a buried object observed at 1,468 m in a relatively flat area located more than 3 km to the north-northeast of the canyon (Figs 3 and 4).

**Fig 5:**
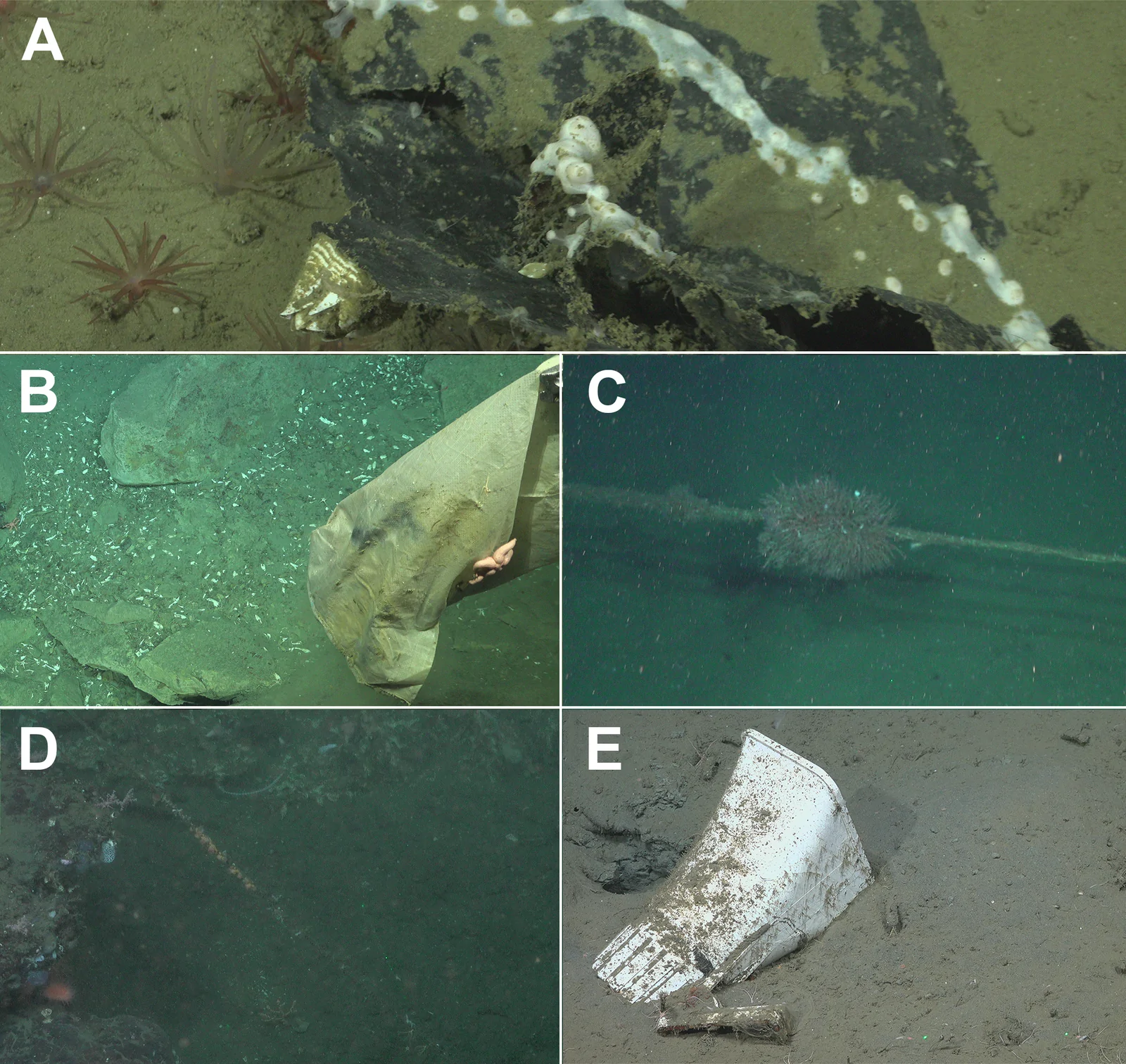
Faunal interactions with anthropogenic litter in the Mar del Plata Submarine Canyon (MPSC). **Dive identification codes (e.g., S0814, S0819) correspond to ROV SuBastian dives.** (A) Degraded black plastic sheet, partially buried, with arthropods including *Tetrachaelasma southwardi* and an octocoral (S0814 - event 4), 2,108 m. (B) Plastic bag with the asteroid *Smilasterias* sp. (S0819 - event 18), 3,243 m. (C) Fishing rope with tubicolous polychaetes (S0814 - event 8), 2,054 m. (D) Nylon filament (probably fishing line) with Anthozoa polyp indet. (S0814 - event 10), 1,616 m. (E) White plastic container, partially buried, with the brittle star *Ophioplinthus* sp. and probable ascidian (S0816 - event 15), 1,764 m. All images courtesy of Schmidt Ocean Institute/ ROV SuBastian (CC BY 4.0), expedition FKt250712.

Marine litter was not evenly distributed throughout the canyon but instead occurred in several localized accumulation zones. The largest concentration was observed in the upper sector of the canyon, where the valley is deeply incised (down to around 2,000–2,100 m depth) and 800 m wide. In the upper sector, 10 items were found clustered along two dive transects (S0814 and S0818) between 1,700 and 2,000 m depth (Fig 4A). Most of the fishing-related items (3 out of 4) were found in this area (Table 1, S1 Table), along with three buried or degraded plastic items, one piece of drifting plastic, and three objects lying on the seabed (Table 1, S1 Table, Fig 2C). Two of the fishing-related items observed in this sector of the canyon, including one relatively long rope, were located on the northern wall (Table 1, S1 Table, Fig 5C-D), while the third was found on the southern wall (Table 1, Fig 2H). The remaining items were recorded between the base of the northern wall and the center of the valley (250–300 m from the wall base) (Table 1, S1 Table, Fig 2A). During dive S0814, nine litter items were observed along a 3.14 km transect, corresponding to fewer than three items per kilometer of ROV track.

A second accumulation zone was identified at approximately 2,500 m depth, where the canyon widens to around 1,200 m (Fig 4B). Four litter items were documented in this sector, including two degraded or buried items on the northern wall (1,700–1,800 m) and an Asian-origin food wrapper. A rubber boot (manufactured in Argentina) was collected from the valley floor at a depth of 2,047 m, approximately 400 m from the base of the northern wall (Fig 2B).

In the lower sector of the canyon, between 3,100 and 3,200 m, five litter items were concentrated on the southern wall near the confluence of a tributary valley and the main canyon (Fig 4C). Three items (a plastic cup, a plastic bag, and a snack wrapper) were trapped among rocks and boulders, forming a localized litter deposit (Fig 2D, 2I). A large bag and a yellow rubber work glove were also collected nearby. At the exit of the tributary valley, 2.5 km east of this group, a heavily degraded black plastic bag was observed at 3,075 m (Table 1, S1 Table).

A fourth cluster of five items occurred in the deepest surveyed section of the canyon (~3,700 m), where the valley is about 2 km wide. Observations included a sediment-filled bag at 3,739 m (Fig 2F), three buried or degraded items, and a green nylon strip between 3,600 and 3,700 m depth, at the base of the northern wall (Table 1, S1 Table, Figs 2G and 4D).

The deepest item recorded in the MPSC was an isolated tissue-paper wrapper (>10 cm) at a depth of 3,814 m near the center of the canyon valley. At this depth, the valley exceeds 10 km in width, and its morphology gradually becomes less defined as the canyon approaches its terminus (Fig 2E).

### Benthic fauna associated with marine litter

**Fig 6:**
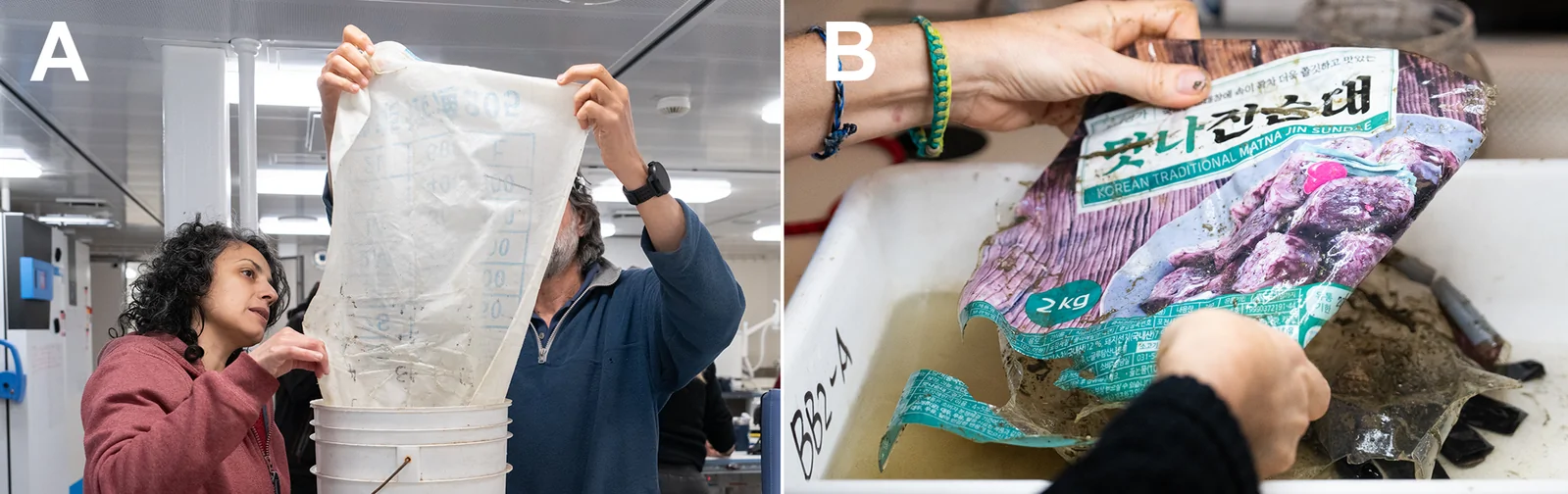
Collected litter for examination purposes in the Mar del Plata Submarine Canyon (MPSC) (Ph. **Misha Vallejo/ Schmidt Ocean Institute). Dive identification codes (e.g., S0814, S0819) correspond to ROV SuBastian dives.** (A) Large plastic bag from S0812 - event 1, 1,120 m. (B) Medium-sized plastic wrapper from S0814 - event 3, 2,109 m. Reproduced with permission from Schmidt Ocean Institute, original copyright 2025.

Deep-sea benthic organisms were observed associated with seven of the 29 litter items. (Table 1, S1 Table, Fig 5). Associated taxa included asteroids, ophiuroids, crustaceans, cnidarians, polychaetes, and probable ascidians. Observed litter-fauna interactions included both sessile colonization (habitat provision) (Table 1, Fig 5A, C-D) and associations with mobile fauna that can also occur independently of litter (encountering) (Table 1, S1 Table, Fig 5A-5B, 5E). Fauna were associated with a variety of litter, including intact or partially buried plastic bags, highly degraded bags, plastic containers, and nylon fishing gear. Of the seven items bearing associated fauna, five were plastic bags or wrappers that were partially buried in the sediment, while the remaining two were fishing-related gear (rope and filament). No additional fauna were observed on the litter items recovered and examined onboard (Fig 6).

## Discussion

The predominance of plastics and fishing-related debris in the MPSC is consistent with reported patterns in deep-sea environments worldwide [4,6,8,9,12,15,38,39]. In many regions, deep-sea litter is dominated by materials associated with fishing activities, including lost or discarded gear and food packaging, reflecting the influence of offshore fisheries operating along continental margins [8]. Similar patterns have recently been documented in the widerArgentine deep sea, where plastic debris and fishing-related items were the dominant litter categories [20].

The composition of litter in the MPSC suggests a predominantly maritime origin. The canyon is land-detached and located more than 250 km offshore, with no direct connection between the continental shelf and the canyon head [22,39]. Consequently, direct inputs from coastal urban sources are likely to be limited. Instead, litter may originate from offshore activities above or adjacent to the canyon and subsequently accumulate within the canyon through downslope transport and topographic trapping [8,9]. Direct disposal associated with fishing operations may also contribute to the observed accumulation of litter. The greater concentration of litter along the northern canyon wall further suggests that local circulation patterns, including the northward-flowing Malvinas Current [26], may influence litter transport and retention within the canyon.

Benthic fauna were associated with only a subset of the litter items observed, suggesting that the stability and residence time of anthropogenic debris on the seafloor influence the likelihood of colonization. In the present observations, organisms were primarily associated with partially buried plastic bags and with fishing-related materials such as ropes and nylon filaments. Partial burial likely stabilizes otherwise mobile plastic items and indicates prolonged exposure on the seafloor, thereby increasing opportunities for colonization by benthic organisms. In contrast, associations with mobile fauna are likely more transient and reflect behaviors such as shelter-seeking, feeding, or exploration [1,4,8,13]. Similar litter–fauna associations have been reported in other deep-sea environments, where litter can serve as an artificial hard substrate in soft-sediment-dominated habitats [6,10,40]. The persistence and accumulation of such litter may facilitate the development of epibenthic assemblages on these artificial substrates [17]. In addition, mobile plastic bags have been reported to transport egg capsules and other organisms, potentially facilitating dispersal to distant environments [13].

Although several litter items appeared to support benthic fauna, these associations should not be interpreted as evidence that anthropogenic litter constitutes a functional or beneficial component of deep-sea ecosystems. Rather, they illustrate the capacity of persistent artificial material to become incorporated into natural ecological processes. Marine litter remains a chronic anthropogenic disturbance that can alter habitat structure, modify ecological interaction, and potentially polluting materials into ecosystems that have largely evolved in their absence [41–43].

ROVs provide a powerful and minimally invasive approach for investigating marine litter, enabling remote exploration of deep and topographically complex environments that are otherwise difficult to access. High-resolution imaging systems facilitate the accurate documentation, characterization, and spatial mapping of litter items [9,10]. Nevertheless, because this study relies exclusively on visual records, the reported observations likely underestimate the true extent of litter on the seafloor, as small, partially buried, or obscured items may have remained undetected [8]. Despite this limitation, the occurrence of marine litter throughout the MPSC, including at depths exceeding 3,800 m, demonstrates the far-reaching influence of human activities on remote deep-sea ecosystems. These observations further suggest that offshore operations, particularly fishing activities, may contribute to the transport and accumulation of litter in remote deep-sea environments and highlight the need for improved management, monitoring, and retrieval of fishing gear and other materials used in these areas.

Submarine canyons are recognized as hotspots for marine litter accumulation (see [17] for a review). However, most studies have focused on land-attached canyons, where canyon heads intersect the continental shelf or occur adjacent to densely populated coastlines, such as in the Mediterranean Sea (e.g., the canyons off the coast of Sicily and Sardinia) and on the west coast of the USA (e.g., the Monterey Canyon). In these systems, litter inputs are strongly influenced by rivers, coastal runoff, and shelf transport processes and litter density can be as high as 50–80 items/km [17].

In contrast, land-detached canyons have received little attention with respect to marine litter because they are generally considered inactive and not affected by the currents that carry litter into their valleys [17]. Our study demonstrates the presence of litter items in a land-detached canyon (Mar del Plata), whose head is located several hundred kilometres from the sparsely populated Mar del Plata town (which has fewer than 700,000 inhabitants). Although litter densities in the MPSC appear substantially lower than those reported for many Mediterranean and North American canyon systems (litter density for dive S0814 in MPSC was less than 3 items/km), anthropogenic debris was nevertheless widespread and formed localized hotspots of accumulation within the canyon. In other areas of the open Argentine Sea, the occurrence of litter was even lower (0.3–1.4 items per km; 20). The size of the Argentine continental shelf, the distance between the canyon head and the coast, and the fact that the MPSC is detached from land all contribute to the relatively low, yet still concerning, amount of litter on the seafloor. The predominance of plastic materials and the presence of fishing-related debris suggest that offshore activities, particularly high-seas fisheries, are important contributors to litter inputs in this remote deep-sea environment.

These findings highlight the need for long-term monitoring and targeted conservation measures in deep-sea environments. By establishing a valuable baseline for the occurrence and distribution of marine litter in the MPSC, this study provides a foundation for future assessments of anthropogenic impacts and for the development of policies aimed at reducing marine litter from multiple sources at regional and global scales.

## Supporting information

S1 Table Classification of marine litter items recorded during ROV dives in the Mar del Plata Submarine Canyon, including dive identification code, sampling site (CM: Contourite moat; NCW: Northern canyon wall; SCW: Southern canyon wall; CV: canyon valley), water depth (m), litter category and description, substrate, associated fauna, and video reference. (DOCX)
