# Supplementary material for: Out of sight, hidden into the deep: Marine litter in the Mar del Plata Submarine Canyon (southwestern Atlantic) revealed by ROV survey
Source: PLoS One. 2026 Sep 16;21(9):e0357098. doi: 10.1371/journal.pone.0357098 (PMC13581013; doi:10.1371/journal.pone.0357098)
Supplement: Supplementary file 1 [file pone.0357098.s001.docx]

**S1 Table**. **Classification of marine litter items recorded during ROV dives in the Mar del Plata Submarine Canyon, including dive identification code, sampling site (CM: Conturite moat; NCW: Northern canyon wall; SCW: Southern canyon wall; CV: canyon valley), water depth (m), litter category and description, substrate, and associated fauna, and video reference.**

| **Item** | **Dive** | **Site** | **Time** | **Date** | **Lat. (S)** | **Long. (W)** | **Depth** | **Litter Category (MSFD)** | **Litter description** | **Substrate** | **Associated fauna** | **Link to YouTube** |
| --- | --- | --- | --- | --- | --- | --- | --- | --- | --- | --- | --- | --- |
| 1 | S0812 | CM | 20:48:58 | 7/26/25 | 37.971523 | 54.410487 | 1120 | Plastic | Large whitish bag tied with a blue nylon cord. Sampled | Mixed | **Phylum Cnidaria**: *Bathelia candida* (Family: Oculinidae), *Thouarella viridis* (Family: Primnoidae). | <https://www.youtube.com/watch?v=w4XPAlruDfw&list=PLJGVqQI3okzajYuncKPPr3AwDA82-F6fr&index=46> |
| 2 | S0814 | NCW | 1:13:02 | 7/28/25 | 37.870566 | 54.171045 | 2105 | Plastic | Small wrapper drifting near the seafloor | Water column | - | <https://www.youtube.com/watch?v=14O-6nAVzR8&list=PLJGVqQI3okzajYuncKPPr3AwDA82-F6fr&t=21m59s> |
| 3 | S0814 | NCW | 1:36:50 | 7/28/25 | 37.870542 | 54.170896 | 2109 | Plastic | Medium-sized wrapper resting on the seafloor. Label: “Korean traditional matna jin sundae” (sausages). Sampled | Soft bottom | - | <https://www.youtube.com/watch?v=14O-6nAVzR8&list=PLJGVqQI3okzajYuncKPPr3AwDA82-F6fr&t=45m46s> |
| 4 | S0814 | NCW | 2:06:52 | 7/28/25 | 37.870689 | 54.170926 | 2108 | Plastic | Black sheet, highly degraded. partially buried in the sediment | Soft bottom | **Phylum Arthropoda**: Crustacea, Peracarida: Stenetriidae, Munnopsidae (Isopoda), Caprellidae (Amphipoda). Cirripedia: *Tetrachaelasma southwardi* (Bathylasmatidae), and Scalpellomorpha. **Phylum Cnidaria**:  Class Octocorallia, Class Hydrozoa (Family Candelabridae). | <https://www.youtube.com/watch?v=14O-6nAVzR8&list=PLJGVqQI3okzajYuncKPPr3AwDA82-F6fr&t=1h13m10s> |
| 5 | S0814 | NCW | 2:28:18 | 7/28/25 | 37.870767 | 54.171008 | 2108 | Plastic | Snack wrapper | Soft bottom | - | <https://www.youtube.com/watch?v=14O-6nAVzR8&list=PLJGVqQI3okzajYuncKPPr3AwDA82-F6fr&t=1h37m10s> |
| 6 | S0814 | NCW | 3:22:39 | 7/28/25 | 37.870566 | 54.170497 | 2105 | Plastic | Black bag fragment resting on the sediment | Soft bottom | - | <https://www.youtube.com/watch?v=14O-6nAVzR8&list=PLJGVqQI3okzajYuncKPPr3AwDA82-F6fr&t=2h31m33s> |
| 7 | S0814 | NCW | 3:50:36 | 7/28/25 | 37.870456 | 54.170249 | 2101 | Plastic | Greenish sheet buried in the sediment | Soft bottom | - | <https://www.youtube.com/watch?v=14O-6nAVzR8&list=PLJGVqQI3okzajYuncKPPr3AwDA82-F6fr&t=2h59m34s> |
| 8 | S0814 | NCW | 5:29:17 | 7/28/25 | 37.869775 | 54.168389 | 2054 | Plastic | Rope with communities of tubicolous polychaetes | Soft bottom | **Phylum Annelida**: Tubicolous polychaetes. | <https://www.youtube.com/watch?v=14O-6nAVzR8&list=PLJGVqQI3okzajYuncKPPr3AwDA82-F6fr&t=4h38m15s> |
| 9 | S0814 | NCW | 6:06:12 | 7/28/25 | 37.869683 | 54.167929 | 2047 | Plastic | Wrapper fragment with Asian characters | Soft bottom | - | <https://www.youtube.com/watch?v=14O-6nAVzR8&list=PLJGVqQI3okzajYuncKPPr3AwDA82-F6fr&t=5h15m14s> |
| 10 | S0814 | NCW | 13:11:22 | 7/28/25 | 37.868023 | 54.162741 | 1616 | Plastic | Nylon filament | Mixed | **Phylum Cnidaria**: Anthozoa polyp indet. | <https://www.youtube.com/watch?v=qt22uyRZqnM&list=PLJGVqQI3okzajYuncKPPr3AwDA82-F6fr&t=5h16m12s> |
| 11 | S0815 | NCW | 2:43:43 | 7/29/25 | 37.819521 | 54.096115 | 1468 | Plastic | Indeterminate black rectangular fragment | Mixed | - | <https://www.youtube.com/watch?v=xwhBVU6IzEo&list=PLJGVqQI3okzajYuncKPPr3AwDA82-F6fr&t=6h2m26s> |
| 12 | S0816 | NCW | 5:08:19 | 7/30/25 | 37.873279 | 54.066246 | 1920 | Plastic | Wrapper with Asian characters – Indonesian fried noodles (Mi goreng) | Mixed | - | <https://www.youtube.com/watch?v=wPeAwx1pUKU&list=PLJGVqQI3okzajYuncKPPr3AwDA82-F6fr&t=6h8m18s> |
| 13 | S0816 | NCW | 5:10:28 | 7/30/25 | 37.873191 | 54.066226 | 1913 | Plastic | Transparent, highly degraded,  bag partially buried in the sediment | Mixed | **Phylum Arthropoda**: Crustacea, Peracarida: probably Asellota (Isopoda). | <https://www.youtube.com/watch?v=wPeAwx1pUKU&list=PLJGVqQI3okzajYuncKPPr3AwDA82-F6fr&t=6h10m23s> |
| 14 | S0816 | NCW | 14:35:34 | 7/30/25 | 37.896283 | 54.053459 | 2598 | Rubber | Black boot with whitish soil, labeled “Design Italy, Industria Argentina”). Sampled | Soft bottom | - | <https://www.youtube.com/watch?v=bbE8Hw9ClC0&list=PLJGVqQI3okzajYuncKPPr3AwDA82-F6fr&t=17m7s> |
| 15 | S0816 | NCW | 7:26:57 | 7/30/25 | 37.868427 | 54.062138 | 1764 | Plastic | White container, partially buried in the sediment | Soft bottom | **Phylum Echinodermata**: *Ophioplinthus* sp. (Family Ophiopyrgidae). **Phylum Chordata**: Probably ascidian | <https://www.youtube.com/watch?v=wPeAwx1pUKU&list=PLJGVqQI3okzajYuncKPPr3AwDA82-F6fr&t==8h26m59s> |
| 16 | S0818 | NCW | 9:18:30 | 8/1/25 | 37.886965 | 53.869687 | 2165 | Plastic | Whitish  anthropogenic strip | Soft bottom | - | <https://www.youtube.com/watch?v=Fa-iwwxiDr0&list=PLJGVqQI3okzajYuncKPPr3AwDA82-F6fr&t=10h45m15s> |
| 17 | S0819 | SCW | 20:32:00 | 8/1/25 | 37.940149 | 53.904447 | 3243 | Plastic | White cup | Mixed with big boulders | - | <https://www.youtube.com/watch?v=FJNJJ8L8Vwo&list=PLJGVqQI3okzajYuncKPPr3AwDA82-F6fr&t=3h24m43s> |
| 18 | S0819 | SCW | 20:32:00 | 8/1/25 | 37.940149 | 53.904447 | 3243 | Plastic | Whitish bag with attached sea star. Sampled | Mixed with big boulders | **Phylum Echinodermata**: *Smilasterias* sp. (Family Asteriidae). | <https://www.youtube.com/watch?v=FJNJJ8L8Vwo&list=PLJGVqQI3okzajYuncKPPr3AwDA82-F6fr&t=3h24m43s> |
| 19 | S0819 | SCW | 23:42:28 | 8/1/25 | 37.940386 | 53.904669 | 3231 | Plastic | Snack wrapper | Mixed with big boulders | - | <https://www.youtube.com/watch?v=FJNJJ8L8Vwo&list=PLJGVqQI3okzajYuncKPPr3AwDA82-F6fr&t=6h34m38s> |
| 20 | S0819 | SCW | 4:21:02 | 8/2/25 | 37.940936 | 53.904658 | 3110 | Plastic | Whitish large bag | Mixed | - | <https://www.youtube.com/watch?v=kAxqt4UqdLo&list=PLJGVqQI3okzajYuncKPPr3AwDA82-F6fr&t=3h2m27s> |
| 21 | S0819 | SCW | 11:48:37 | 8/2/25 | 37.943666 | 53.902640 | 3078 | Rubber | Yellow glove | Hard bottom with sediments | - | [https://www.youtube.com/watch?v=kAxqt4UqdLo&list=PLJGVqQI3okzajYuncKPPr3AwDA82-F6fr&t=10h30m6s](https://www.youtube.com/watch?v=kAxqt4UqdLo&list=PLJGVqQI3okzajYuncKPPr3AwDA82-F6fr&it=10h30m6s) |
| 22 | S0820 | NCW | 12:34:55 | 8/2/25 | 38.056873 | 53.669210 | 3739 | Plastic | Green nylon strip. Sampled | Soft bottom | - | <https://www.youtube.com/watch?v=lcM82Srz8TE&list=PLJGVqQI3okzajYuncKPPr3AwDA82-F6fr&t=14m12s> |
| 23 | S0820 | NCW | 13:02:16 | 8/4/25 | 38.056772 | 53.669042 | 3739 | Plastic | Sediment-filled bag | Soft bottom | - | <https://www.youtube.com/watch?v=lcM82Srz8TE&list=PLJGVqQI3okzajYuncKPPr3AwDA82-F6fr&t=41m28s> |
| 24 | S0820 | NCW | 16:41:22 | 8/4/25 | 38.054698 | 53.667927 | 3722 | Plastic | Whitish sheet or bag fragment, highly degraded | Soft bottom | - | <https://www.youtube.com/watch?v=lcM82Srz8TE&list=PLJGVqQI3okzajYuncKPPr3AwDA82-F6fr&t=4h20m43s> |
| 25 | S0820 | NCW | 16:51:29 | 8/4/25 | 38.054515 | 53.667855 | 3720 | Plastic | Whitish sheet or bag fragment, highly degraded | Soft bottom | - | <https://www.youtube.com/watch?v=lcM82Srz8TE&list=PLJGVqQI3okzajYuncKPPr3AwDA82-F6fr&t=4h30m42s> |
| 26 | S0820 | NCW | 18:12:53 | 8/4/25 | 38.053695 | 53.667506 | 3709 | Plastic | Whitish fragment, highly degraded | Soft bottom | - | <https://www.youtube.com/watch?v=lcM82Srz8TE&list=PLJGVqQI3okzajYuncKPPr3AwDA82-F6fr&t=5h52m5s> |
| 27 | S0821 | AP | 2:52:54 | 8/6/25 | 38.150488 | 53.428942 | 3814 | Plastic | Blue printed tissue paper wrapper | Soft bottom | - | <https://www.youtube.com/watch?v=hVc5bTguGyc&list=PLJGVqQI3okzajYuncKPPr3AwDA82-F6fr&t=29m40s> |
| 28 | S0823 | SCW | 3:46:31 | 8/8/25 | 37.940608 | 53.934125 | 3075 | Plastic | Highly degraded black fragment | Soft bottom | - | <https://www.youtube.com/watch?v=DBhPSqv0x20&list=PLJGVqQI3okzajYuncKPPr3AwDA82-F6fr&t=3h52m14s> |
| 29 | S0825 | SCW | 10:48:10 | 8/9/25 | 37.881749 | 54.175258 | 1949 | Plastic | Nylon rope with a hook. Sampled | Hard bottom | - | <https://www.youtube.com/watch?v=ViB0Vm4wxqU&list=PLJGVqQI3okzajYuncKPPr3AwDA82-F6fr&t=7h49m3s> |
